# Impact of Neuropathy on Well-Being and Health-Related Quality of Life in Adolescents With Type 1 Diabetes

**DOI:** 10.1155/jdr/6620727

**Published:** 2025-04-17

**Authors:** Vinni Faber Rasmussen, Mathilde Thrysøe, Páll Karlsson, Jens Randel Nyengaard, Kurt Kristensen, Esben Thyssen Vestergaard

**Affiliations:** ^1^Department of Pediatrics and Adolescents, Randers Regional Hospital, Randers, Denmark; ^2^Aarhus University, Aarhus, Denmark; ^3^Danish Pain Research Center, Department of Clinical Medicine, Aarhus University, Aarhus, Denmark; ^4^Core Centre for Molecular Morphology, Section for Stereology and Microscopy, Department of Clinical Medicine, Aarhus University, Aarhus, Denmark; ^5^Department of Pathology, Aarhus University Hospital, Aarhus, Denmark; ^6^Steno Diabetes Center Aarhus, Aarhus University Hospital, Aarhus, Denmark; ^7^Department of Pediatrics and Adolescents, Aarhus University Hospital, Aarhus, Denmark

## Abstract

**Aim:** This study is aimed at assesing the impact of neuropathy on well-being and health-related quality of life (HRQoL) in adolescents with Type 1 diabetes (T1D).

**Methods:** In a cross-sectional study, 60 adolescents with T1D (15–18 years, diabetes duration > 5 years) were enrolled. Clinical and biochemical data were collected, and well-being and HRQoL were assessed using the WHO-5 well-being index and DISABKIDS questionnaires, including diabetes-specific modules. Diagnostic tests for large fiber neuropathy (LFN), small fiber neuropathy (SFN), and autonomic neuropathy were performed as part of the T1DANES study. The participants were divided into groups depending on the presence or absence of specific forms of neuropathy. Those with autonomic neuropathy were further divided depending on reported autonomic symptoms (Composite Autonomic Symptom Scale 31 (COMPASS-31) score ≥ 24 or < 24). Additionally, the data was compared to 23 healthy control subjects.

**Results:** The median diabetes duration was 8.5 years (range 5–17), and the HbA1c was 60 mmol/mol (7.6%) (range 41–93 [5.9%–10.6%]). Adolescents who had abnormal autonomic function test(s) and a COMPASS-31 score ≥ 24 exhibited lower WHO-5 well-being index compared to the following groups: those with abnormal autonomic test(s) and fewer autonomic symptoms (COMPASS-31 < 24), the remaining adolescents with T1D, and the control subjects (*p* values < 0.05). There was no significant difference in the total score of DISABKIDS between the groups; however, the subdomain *social inclusion* was lowest in the group with COMPASS-31 ≥ 24. Gastric motility index (*p* = 0.04) and uroflow acceleration (*p* = 0.02) were positively associated with the total score of DISABKIDS. Females reported lower well-being and HRQoL than males (*p* values < 0.05); in total, 28% had a WHO-5 well-being index < 50.

**Conclusion:** In conclusion, adolescents with diabetic autonomic neuropathy who also reported autonomic symptoms had lower well-being and impaired social inclusion. Adolescents with symptoms of neuropathy and females appear to be at higher risk of lower well-being, and using standardized screening tools helps to identify the subjects at risk.

## 1. Introduction

Psychosocial problems are common among people with diabetes [1], and a literature review reported that mental health problems were 2.3 times more likely in adolescents with Type 1 diabetes (T1D) as compared to adolescents without diseases [2]. Dietary restrictions, self-monitoring of blood glucose, taking insulin injections, feeling different, and lack of support from family and health care professionals have been attributed as causes of the higher occurrence of psychosocial problems [3]. In addition, diabetes-associated problems such as poor glycemic control and complications are associated with lower quality of life (QoL) [4, 5].

Diabetic peripheral neuropathy impairs QoL [6], especially in people having neuropathic pain [7]. It is unknown whether symptoms related to neuropathy are underdiagnosed due to the challenges of diagnosing nerve damage [8]. Neuropathy can result in varying symptoms, depending on the involved types of nerves. The symptoms include, for example, pain in large fiber damage and loss of temperature sensation in small fiber damage [9]. Cardiovascular, pupillary, sudomotor, gastrointestinal, and genitourinary symptoms are related to autonomic dysfunction, as well as erectile dysfunction in males [10]. Gastrointestinal and urinary tract symptoms are commonly reported in adolescents with T1D. The prevalence of gastrointestinal symptoms has been reported to be as high as 75% in adolescents with diabetes [11], and lower urinary tract symptoms have been found twice as prevalent in children with diabetes than those without (33.3% vs. 16.17%) [12].

It is important to recognize symptoms of neuropathy and mental health issues to prevent deterioration and to attempt to improve glycemic control and diabetes outcomes.

The objective of this study was to investigate well-being and health-related quality of life (HRQoL) in adolescents with T1D and neuropathy using the WHO-5 well-being index and the DISABKIDS questionnaire. We aimed to explore whether different types of neuropathies (large fiber neuropathy (LFN), small fiber neuropathy (SFN), and autonomic neuropathy) affect QoL. We hypothesized that the symptomatic presentation of these neuropathies could negatively impact well-being and HRQoL.

## 2. Methods

### 2.1. Study Population

The study was cross-sectional and a part of the T1DANES study [13]. In short, adolescents aged 15–18 years with T1D and a history of diabetes for at least 5 years were recruited among patients attending the outpatient clinics at the hospitals in Randers, Aalborg, and Steno Diabetes Center Aarhus in Denmark between August 2020 and December 2021. Exclusion criteria were participants who had other diseases and other medications than insulin that could affect the central or peripheral nervous system. The presence of associated well-controlled autoimmune disorders (i.e., thyroid disease and celiac disease) or complications to diabetes (i.e., microalbuminuria) were not among the exclusion criteria.

Information about age, gender, diabetes duration, total daily insulin dose, basal insulin dose, time-in-range, glucose-monitoring system, HbA1c values for the last 5 years, events of severe hypoglycemia and ketoacidosis during the last year, and the latest test results for retinopathy and nephropathy (urine albumin/creatinine ratio) were obtained from the patients' clinical electronic files.

Informed oral and written consent was obtained from each participant and the legal guardian. All procedures in the study protocol were approved by the Danish Ethics Committee (Project ID M-2019-211-19) and Legal Office, Central Denmark Region (1-16-02-42-21). Data were stored in REDCap administrated by Aarhus University (REDCap 12.0.30-2023 Vanderbilt University), which is a secure web application for online surveys and databases.

### 2.2. Clinical and Biochemical Data Collection

All participants underwent a single test day at Aarhus University Hospital. In cases where data was missing from the patients' clinical electronic files, supplemental data was collected during the visit.

The weight and height of each participant were measured, and BMI was calculated as weight/height [2] in kilograms and meters. Blood pressure was recorded by an automatic blood pressure monitor.

### 2.3. Questionnaires

Electronic questionnaires were answered at home within the 2 weeks before the test day and directly stored in REDCap. The included validated questionaries were (1) WHO-5 well-being index: a 5-item instrument reflecting the mental well-being during the last 2 weeks [14], (2) DISABKIDS chronic–generic questionnaire measuring the HRQoL including the diabetes-specific module (DSM-10) [15], and (3) Composite Autonomic Symptom Scale 31 (COMPASS-31) evaluating the presentation of autonomic symptoms [16].

Table 1 describes the included questionnaires about HRQoL and well-being. A higher score for both questionnaires indicates higher well-being. When the WHO-5 is used for depression screening, a cut-off score of ≤ 50 has been employed, while a WHO-5 index ≤ 28 is found to be more restrictive and equivalent to the level of well-being observed in patients with depression [14].

### 2.4. Diagnostic Test Indicating Neuropathy

The neurological tests assessing neuropathy are described in separate publications as offspring of the T1DANES study [13, 17, 18], which included 60 adolescents with T1D and 23 healthy control subjects. The diagnostic tests included nerve conduction studies (NCSs) for LFN and intraepidermal nerve fiber density (IENFD) estimated in skin biopsies for SFN. The autonomic function tests included cardiovascular reflex tests (CARTs), tilt table tests, quantitative sudomotor axon reflex tests (QSARTs), wireless motility capsules, and uroflowmetry. Participants were categorized as having autonomic neuropathy if they had one or more abnormal autonomic test(s).

In summary, the definitions of abnormalities of the different included tests were as follows:

- NCS: Abnormal result if ≥ 2 different nerves (motoric and/or sensory) had abnormal outcomes, which include conduction velocity, sensory and motor action potential amplitudes, distal motor latencies, and minimum *F*-wave latencies.
- IENFD: Abnormal result if IENFD < 4.

Autonomic tests:

- CARTs: Abnormal result if ≥ 2 abnormal findings from the HR responses during the following tests: (1) deep breathing with a calculation of the difference between HR during expiration and inspiration, (2) forcefully exhaling with an expiratory pressure of 40 mmHg for 15 s in a 20° tilt position and calculation of the Valsalva maneuver (VM) ratio, and (3) position change to standing and calculation of the 30:15 ratio.
- QSARTs: Abnormal result if the participant had a reduced sudomotor volume at the foot and a length-dependent decrease, which was defined as a sudomotor volume at the foot less than one-third of the volume at the proximal site.
- Orthostatic hypotension: Abnormal result if the participants had a reduction of at least 20 mmHg in systolic BP or 10 mmHg in diastolic BP within 3 min of head-up tilt-table testing.
- Wireless motility capsule: Abnormal result if prolonged transit time and/or lowered motility index.
- Uroflowmetry: Abnormal result if the curve was not bell-shaped and/or reduced uroflow acceleration.

The cut-off for abnormalities was based on the data < 5th percentile or > 95th percentile of the normative data set obtained from healthy adolescents in the T1DANES study.

The Toronto criteria were used to classify individuals into subclinical and confirmed categories for both LFN and/or SFN [19].

The participants were divided into five groups:

- Group 0, T1D, no neuropathy
- Group 1, T1D, confirmed LFN and/or SFN
- Group 2, T1D, subclinical LFN
- Group 3, T1D, subclinical SFN
- Group 4, T1D, abnormal autonomic functional test(s)
  o. 4a: abnormal autonomic functional test(s)+COMPASS-31 ≥ 24
  o. 4b: abnormal autonomic functional test(s)+COMPASS-31 < 24
- Group 5, T1D, mixed: abnormal autonomic functional test(s)+any types of LFN and/or SFN

The cut-off for COMPASS-31 to indicate autonomic symptoms was set to ≥ 24, corresponding to the third quartile of healthy controls in the T1DANES study [13].

The secondary subdivisions of the participants include

- presence or absence of confirmed LFN/SFN
- presence or absence of abnormal autonomic test(s)+COMPASS-31 ≥ 24

### 2.5. Statistical Analysis

All statistical analyses were performed in the software program R (R Core Team (2022), Vienna, Austria). All groups of variables were tested for normal distribution by the Shapiro–Wilk test and QQ-plots. Descriptive data are presented as median (SD) for continuous normally distributed data, median (range) for continuous nonparametric data, and number (percent) for categorical variables, respectively. Comparisons were investigated by Student's *t*-test for continuous variables with normal distribution, Wilcoxon rank-sum test for nonparametric continuous variables, and Fisher's exact test for categorical variables. *p* values < 0.05 were considered significant for all data analyses, and in the T1DANES study, we estimated that our sample size would comply with this. Missing data were handled using data removal. R was used to calculate the effect size (Hedges' *g*). Linear regression and Pearson's product-moment correlation in R were used to analyze the associations between parameters.

## 3. Results

### 3.1. Study Population

**Figure 1:**
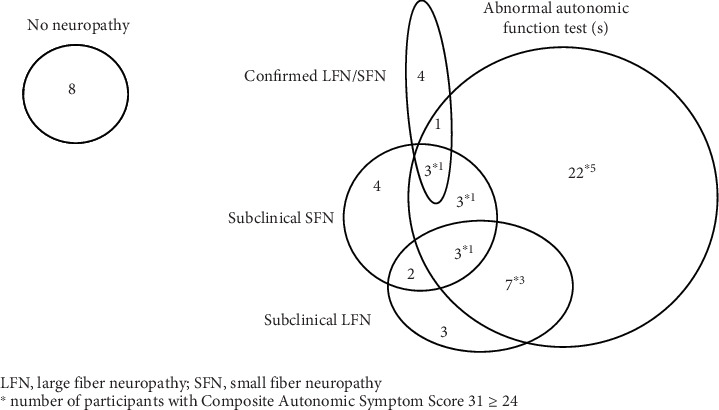
Adolescents with Type 1 diabetes and illustration of the number of participants in each group depending on types of neuropathy.

Sixty adolescents aged 15–18 years with T1D were included with a median diabetes duration of 8.5 years, and a median HbA1c of 60 mmol/mol [7.6%]. Eight had all diagnostic tests for neuropathy normal, and the remaining participants were split into groups depending on types of neuropathy as illustrated in Figure 1. None of the adolescents had symptoms in the length-dependent area, meaning that “confirmed LFN and/or SFN” resulted from abnormal bilateral findings on the neurological examination and abnormal diagnostic tests (abnormal NCS or reduced IENFD). The abnormal clinical findings include reduced reflexes, reduced ability to feel a vibration for 10 s, and reduced ability to feel warm/cold. Only one adolescent had confirmed SFN, and this participant also had confirmed LFN. Therefore, the group for confirmed neuropathy was merged.

Table 2 includes clinical and biochemical characteristics for each group. Notably, Group 4a with abnormal autonomic test(s)+COMPASS-31 ≥ 24 had a large percentage of females (82%) in comparison to the other groups, where females make up between 33% and 53% of the groups.

### 3.2. Results of Well-Being Assessed by the WHO-5 Well-Being Index

**Figure 2:**
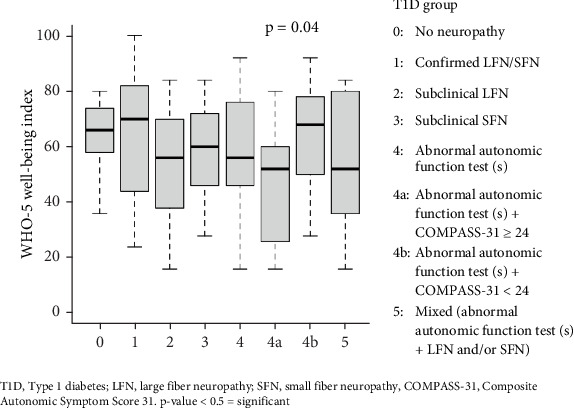
WHO-5 well-being Index in adolescents with Type 1 diabetes depending on the presentation of neuropathy. Data is shown in boxplots. The median is shown as the thick line in the center of the box, the interquartile range (IQR) is shown as the box, and the whiskers extend to the range within 1.5 ⁣^∗^ IQR from the quartiles and end at the minimum and maximum values of the data set.

The median (range) score of the WHO-5 well-being index for all participants with T1D included in the study was 60 (16–100).  Figure 2 shows the WHO-5 index in the different groups. Participants with abnormal autonomic function test results and a COMPASS-31 score ≥ 24 exhibited lower well-being index scores compared to the other groups, significantly to those who reported fewer autonomic symptoms (Group 4a vs. Group 4b; *p* = 0.04, effect size −0.88).

Comparison between participants with T1D with confirmed LFN/SFN (*n* = 8) and those without confirmed LFN/SFN (*n* = 52) revealed no difference in the WHO-5 well-being index and similarly for the comparison between the confirmed LFN/SFN group and the healthy control group (*p* values > 0.05). However, comparison between the participants with abnormal autonomic test(s)+COMPASS-31 ≥ 24 (n = 11) versus the remaining adolescents with T1D (*n* = 49) showed a significant difference in the WHO-5 well-being index (mean score 46 vs. 63, *p* = 0.01, effect size −0.87). Similarly, a significant difference was observed in the WHO-5 well-being index between those with abnormal autonomic test(s)+COMPASS-31 ≥24 and the healthy control group (mean score 46 vs. 62, *p* = 0.03, effect size −0.80).

Dividing the participants with T1D according to sex showed that females had lower WHO-5 well-being scores compared to males (mean score 50 vs. 69, *p* < 0.01, effect size −1.1).

A total of 28% (17/60) of adolescents with T1D had a WHO-5 well-being score < 50, indicating the need for depression screening. Out of all the included adolescents, six individuals (10%) had a WHO-5 index ≤ 28, which is indicative of a stage of depression. All of these individuals were female.

### 3.3. Results of QoL Assessed by DISABKIDS and Diabetes-Specific Domain

The median (range) total score of DISABKIDS for all included adolescents was 136 (75–186).

**Figure 3:**
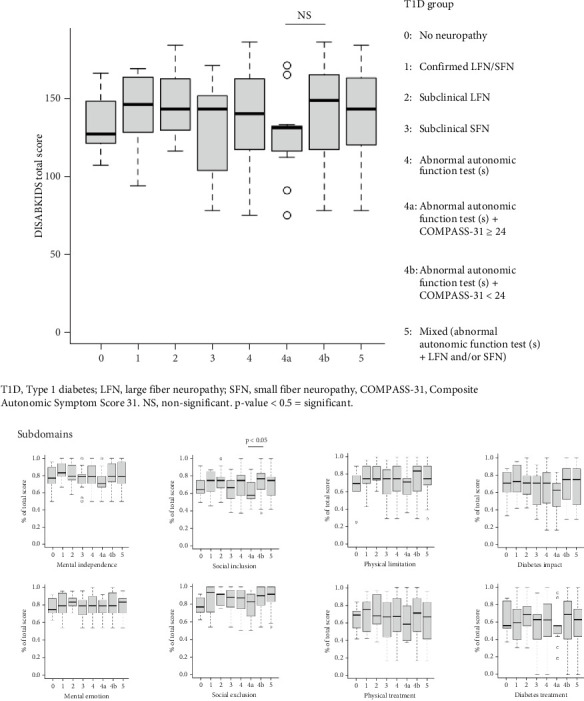
Health-related quality of life in adolescents with Type 1 diabetes depending on the presentation of neuropathy. Data is shown in boxplots. The median is shown as the thick line in the center of the box, the interquartile range (IQR) is shown as the box, and the whiskers extend to the range within 1.5 ⁣^∗^ IQR from the quartiles and end at the minimum and maximum values of the data set. Points outside the whiskers are marked as outliers.

There was no significant difference in the total score of DISABKIDS between the groups, as shown in Figure 3 (all *p* values < 0.05). Similarly, no significant difference in subdomains was observed; however, social inclusion was lower in those with abnormal autonomic functions test(s) and symptoms compared to those expressing fewer autonomic symptoms (Group 4a vs. Group 4b, *p* = 0.045, effect size −0.68). In general, the lowest scores out of the total score in the domain were observed in the physical and diabetes treatment module, as illustrated in Figure 3.

Adolescents with confirmed LFN/SFN were not found to have lower scores of DISABKIDS than the remaining adolescents with T1D (all *p* values > 0.05). Similarly, adolescents with abnormal autonomic test(s)+COMPASS-31 ≥ 24 were not shown to have lower scores of DISABKIDS than the remaining adolescents with T1D (all *p* values > 0.05).

Dividing the participants with T1D according to sex showed that females had lower DISABKIDS scores compared to males (all *p* values < 0.05).

In total, 10% (6/60) of adolescents with T1D had a DISABKIDS score below 50% of the total sum score. Five of the six cases were females, and five of the six cases had “physical limitation” and “social inclusion” below the 50th percentile of the maximum score.

### 3.4. Association Between Well-Being, HRQoL, and Diagnostic Test for Neuropathy

Gastric motility and uroflow are reported in separate articles [17, 18], and when included here as a dependent variable using linear regression analysis, we found that a lower gastric motility index (*r* = 0.30, *p* = 0.04) and a lower uroflow acceleration (*r* = 0.30, *p* = 0.02) were positively associated with the total score of DISABKIDS.

There were no associations between the other neurological tests (conduction velocity of nerves, IENFD, QSART, CARTs, and gastrointestinal transit times) and the results of questionnaires indicating reduced well-being (data not shown, all *p* value > 0.05).

## 4. Discussion

In adolescents with T1D for over 5 years, we showed that those who had abnormal autonomic test(s) and reported more autonomic symptoms in COMPASS-31 exhibited lower well-being and had worse social inclusion. Despite many adolescents with T1D having different types and degrees of neuropathy, no other significant impact on the HRQoL assessed by DISABKIDS was observed compared to those without neuropathy.

It is not surprising that adolescents need to show symptoms of neuropathy before it has a significant impact on their well-being, although the fear of complications may also play a role. The absence of reported symptoms of LFN and SFN among the included adolescents may be attributed to an overall subclinical presentation of these neuropathy forms or a lower impact on bodily functions, although this was not investigated. Furthermore, those with abnormal findings on neurological examination did not exhibit symptoms that significantly impacted their daily lives.

It seems relevant to explore the potential bidirectional relationship between autonomic symptoms and well-being. Research shows that factors such as stress, anxiety, and depression can exacerbate the perception of autonomic symptoms [20]. Conversely, chronic autonomic symptoms may adversely impact well-being by disrupting the autonomic nervous system, which governs vital functions such as heart rate, blood pressure, and digestion, potentially contributing to heightened psychological distress and social debilitation [21]. This complex interplay underscores the importance of addressing both physical and psychological aspects when assessing and managing autonomic symptoms. Interventions targeting psychological well-being may alleviate the perception of symptoms and improve overall health outcomes, also including better metabolic control in adolescents with T1D. Higher HbA1c levels have been linked to increased depressive symptoms and reduced psychosocial well-being [22], emphasizing the crucial role of glucose regulation in supporting the overall well-being of adolescents.

Interestingly, the lowest scores in the subdomains of the DISABKIDS questionnaire were observed in the diabetes module. This suggests that the included adolescents perceive diabetes as disruptive to their lives. They need to carefully consider their dietary choices and have a fear of experiencing blood glucose levels outside of the normal range. Furthermore, a low social score suggests that they feel socially distinct from others, which affects their level of social engagement. Previous studies have shown that adolescents with T1D face greater limitations in activities with friends and schoolwork due to physical challenges [22].

Our study found that females report lower well-being and HRQoL than males, consistent with findings from the WHO's Health Behaviour in School-Aged Children Study, which shows that girls generally report lower well-being [23]. Several potential biological and psychosocial factors may mediate these differences. Biologically, hormonal fluctuations, such as those related to the menstrual cycle, can influence subjective distress [24]. Psychosocial factors, including gender roles, societal expectations, and greater stress or caregiving responsibilities, may also play a role.

Sex was identified as a potential confounder in our study, as the group with abnormal autonomic tests and symptoms included a higher percentage of females. Notably, the EURODIAB Prospective Complications Study identified female sex as a significant risk factor for painful diabetic peripheral neuropathy [25]. There is literature that shows that young women exhibit lower resting sympathetic activity compared with young men [26], further supporting the influence of sex on autonomic function.

More studies focusing on adolescents and sex-specific differences in diabetic neuropathies and well-being are needed to better understand and clarify these findings.

Previously, anxiety symptoms were reported more prevalent in preadolescent males and adolescent females [27]. Our study supported these previous findings in adolescent females, and we also showed that they reported a lower QoL compared to males. Moreover, more females had a critically low score indicating a risk of depression. Our data therefore highlights the importance of paying extra attention to adolescent females with T1D about both their metabolic control and their mental health.

In our study, we observed that lower gastric motility index and lower uroflow acceleration were associated with reduced HRQoL. Diabetic neuropathy may distort the control of intestinal motility. Lower intestinal motility can be caused by multiple factors including damaged parasympathetic nerve fibers, and the condition can lead to diverse symptoms. The questions in DISABKIDS do not explore symptoms associated with autonomic dysfunction. The COMPASS-31 and the GI Symptom Rating Scale (GSRS) can be used for this purpose, and in the T1DANES cohort, associations have been found between the colonic motility index and symptoms of diarrhea and indigestion [17]. Additionally, constipation has been shown to negatively affect the QoL in another population of adolescents [28]. Taken together, the findings suggest that a connection between neuropathic symptoms, diagnostic findings, and QoL cannot be ruled out.

Notably, relying solely on a questionnaire for detecting autonomic neuropathy has inherent limitations in terms of quality [13]. Additionally, it is worth noting that physicians should be mindful that both gastrointestinal and lower urogenital symptoms are also prevalent in healthy children and adolescents, although at a lower frequency [29].

The parasympathetic pudendal nerve innervates the smooth muscles in the bladder wall, and a decreased acceleration of detrusor muscle contraction has been interpreted as an early sign of autonomic neuropathy [30]. Detrusor muscle dysfunction can cause a range of symptoms such as wetting and a constant feeling of bladder fullness, which can lead to discomfort and explain our finding of a positive association between uroflow acceleration and HRQoL, as evaluated with DISABKIDS.

Self-monitoring of blood glucose and taking insulin injections have previously been found to cause psychosocial problems in people with diabetes [3]. Rubin and Peyrot summarized the most frequent issues: hating to monitor blood glucose, fear of taking insulin, the frustration of keeping blood glucose in the normal range, and feeling deprived of food. All these issues lead to a risk of developing depression, anxiety, and eating disorders, which are seen at a higher frequency in adolescents with T1D compared to healthy adolescents [31]. Altogether, this is concerning because depressive and anxiety symptoms lead to poorer diabetes management in youth with T1D [32, 33]. And when knowing that poorer metabolic control is an important risk factor for developing diabetic neuropathy [34], care providers should pay attention to clinical warning signs of psychosocial well-being. Older age in adolescence, higher family conflict, poorer QoL, and higher HbA1c have been described to correlate with psychological problems [33].

Our findings of neuropathy, problematic diabetic impact, and lower well-being are assessed as a “dangerous” risk profile for the progression of neuropathy and related symptoms later in life.

A growing body of evidence indicates that diabetic neuropathy is a risk factor for depression, predicting both the severity and increment in prevalence of depression over time [35]. Consistent with findings in *Dutch adolescents with T1D* [36], our study revealed that approximately a quarter of adolescents had a low well-being score, highlighting the need for depression screening according to recommended guidelines [14]. It seems important to screen already in preadolescents because reduced QoL in patients with diabetic neuropathy is found to start years before their neuropathy diagnosis [37]. More research is needed to identify the optimal screening tool and timing for reduced QoL. However, our best recommended screening tool is the WHO-5 well-being index, as it is simple and quick to administer, validated for use in adolescents with diabetes [38], and widely recognized globally.

Although the WHO-5 is not specifically mentioned in all diabetes management guidelines, assessing mental health and QoL is a key aspect of diabetes care. The ISPAD 2022 Clinical Practice Consensus Guidelines highlight the importance of monitoring HRQoL and psychosocial well-being in children and adolescents with diabetes, including the psychological impact of diabetes management [39]. While not directly specifying the WHO-5, similar tools are crucial for assessing psychosocial effects, aligning with ISPAD's recommendations for regular screening and psychological support. Incorporating the WHO-5 in diabetes care can help clinicians to address the psychological aspects of diabetes management, offering a quick and practical tool for clinical practice.

One limitation of this study is that DISABKIDS is primarily a screening tool, and a practical interpretation of how the results can be utilized and managed is yet to be established. Given the limited literature available on this topic, it challenges comparison, despite the questionnaire being valid and reliable both within and between countries after a few adjustments [40]. In addition, no psychological interviews were conducted to explore a deeper layer of the problems and possible links to symptoms related to diabetes complications. It would have been valuable to include data on potential biases, such as participants' socioeconomic or family backgrounds. A potential bias from self-reporting, including desirability bias, might also exist; however, we attempted to minimize the bias by encouraging the adolescents to complete the questionnaires at home.

Also, the lack of sample size calculation may be mentioned as a limitation. Additionally, our small sample sizes limited the generalizability of the findings. The strength of this study was the use of accepted confirmatory diagnostic tests for neuropathy and that the questionnaires were answered electronically at home without disturbances from hospital environments and care providers.

## 5. Conclusion

In conclusion, adolescents with diabetic autonomic neuropathy and reported autonomic symptoms were shown to have lower well-being and impaired social inclusion. Adolescents with symptoms of neuropathy and females appear to be at higher risk of lower well-being.

The presence of low well-being, impaired HRQoL, and neuropathic symptoms underscores the importance of enhancing screening methods for psychosocial issues and neuropathy early in adolescents with T1D.

## Figures and Tables

**Table 1:** Description of the DISABKIDS and WHO-5 well-being questionnaires.

**Subdomains**		**Items**	**Orientation**	**Max score**	
DISABKIDS (max score 188)					
Mental	Independence	1–6	Neg-pos	24	The items in the questionnaires have five ordinal response categories ranging from “never” to “always.” The scoring of items depends on the orientation of the questions. During the analysis, questions relating to negative experiences were coded 4 (*never*) to 0 (*always*), whereas questions relating to positive experiences were coded 0 (*never*) to 4 (*always*). A high total score, therefore, indicated few problems and a high degree of QoL for all subscales irrespective of the orientation of the items
	Emotion	13–19	Pos-neg	28	
Social	Inclusion	26–31	Neg-pos	24	
	Exclusion	20–25	Pos-neg	24	
Physical	Limitation	7–12	7: neg-pos 8–12: pos-neg	24	
	Treatment	32–37	Pos-neg	24	
Diabetes module	Impact	1–6	Pos-neg	24	
	Treatment	7–10	Pos-neg	16	

WHO-5 (max score 100)					
		1–5	Pos	100	The items in the questionnaires have six ordinal responses ranging from “all of the time” to “at no time.” The questionnaires had a positive orientation. The scoring of items was coded 5 (*all of the time*) to 0 (*at no time*). A high total score indicated a higher degree of QoL.

**Table 2:** Characteristics of the participants with Type 1 diabetes in each group depending on types of neuropathy.

	**No neuropathy**	**Group 1** **Confirmed LFN/SFN**	**Group 2** **Subclinical LFN**	**Group 3** **Subclinical SFN**
*n*	8	8	15	15
Age (years)	17.3 (15.5–17.8)	16.8 (15.7–18.2)	16.5 (15.0–19.0)	16.3 (15.2–18.4)
Sex (female:male)	4:4	3:5	5:10	8:7
Diabetes duration (years)	9.3 (6.5–12.9)	8.9 (5.3–17.4)	8.5 (5.4–14.2)	8.5 (5–17.4)
HbA1c (mmol/mol)	58 (41–77)	67 (43–93)	60 (44–79)	63 (44–81)
Time in range (%)^b^	63 (49–71)	68 (43–76)	47 (23–85)	54 (27–85)
BMI-SDS	0.2 (−1.2 to 1.3)	0.5 (0.4–1.9)	0.6 (−2.3 to 1.6)	0.6 (−0.6 to 1.9)
LDL (mmol/L)	2.2 (1.4–2.8)	2.1 (1.9–4.1)	1.9 (1.4–3.7)	2.0 (0.5–3.3)
Cholesterol (mmol/L)	4.1 (3.1–4.7)	4.0 (3.4–5.5)	4.3 (3.3–6.0)	3.9 (3.0–4.9)
Triglycerides (mmol/L)	0.8 (0.6–1.7)	1.0 (0.6–1.9)	0.9 (0.5–3.8)	0.6 (0.3–2.1)
SysBP (mmHg)	118 (98–130)	115 (103–142)	120 (68–139)	117 (68–142)

	**Group 4** **Abnormal autonomic test(s)**	**Group 4a** **+COMPASS-31** ≥ 24	**Group 4b** **+COMPASS-31 < 24**	**Group 5** **Mixed** ^**a**^
*n*	39	11	28	17
Age (years)	17.0 (15.0–18.8)	16.5 (15.0–18.4)	17.4 (15.2–18.8)	16.7 (15.0–18.6)
Sex (female:male)	19:20	9:2	10:18	6:11
Diabetes duration (years)	8.2 (4.6–17.4)	8.9 (4.7–15.4)	7.9 (4.6–17.4)	8.9 (5.3–17.4)
HbA1c (mmol/mol)	60 (43–93)	58 (52–73)	61 (43–93)	61 (43–79)
Time in range (%)^b^	51 (24–85)	42 (31–67)	52 (24–85)	48 (27–76)
BMI-SDS	0.6 (−0.6 to 1.9)	0.5 (−0.5 to 1.5)	0.6 (−0.6 to 0.9)	0.5 (−0.6 to 1.9)
LDL (mmol/L)	2.0 (0.5–4.1)	1.9 (0.5–4.1)	2.1 (1.0–4.0)	1.9 (0.5–2.8)
Cholesterol (mmol/L)	4.0 (3.0–6.4)	3.8 (3.0–6.2)	4.1 (3.0–6.4)	3.9 (3.0–5.7)
Triglycerides (mmol/L)	0.9 (0.3–3.8)	0.7 (0.3–3.8)	0.9 (0.6–2.1)	0.8 (0.3–3.8)
SysBP (mmHg)	117 (68–147)	109 (68–120)⁣^∗^	120 (102–147)	117 (68–142)

*Note:* Data are presented as median (range).

Abbreviations: COMPASS-31, Composite Autonomic Symptom Scale 31; LFN, large fiber neuropathy; SFN, small fiber neuropathy; sysBP, systolic blood pressure.

^a^Mixed: autonomic abnormal test(s)+LFN and/or SFN.

^b^Only available data of “time in range” from 44 adolescents.

⁣^∗^*p* value < 0.05 (significantly different compared to the group without neuropathy).

## Data Availability

The data sets generated during and/or analyzed during the current study are not publicly available due to the General Data Protection Regulation but are available in an anonymized version from the corresponding author upon reasonable request and acceptance from the Legal Office, Central Denmark Region.
